# Analysis of the Filipinos’ Interest in Searching Online for Oral Cancer

**DOI:** 10.31557/APJCP.2020.21.4.1121

**Published:** 2020-04

**Authors:** Junhel Dalanon, Renelson Esguerra, Liz Muriel Diano, Yoshizo Matsuka

**Affiliations:** 1 *School of Dentistry, Southwestern University PHINMA, Cebu, Philippines, *; 2 *Department of Stomatognathic Function and Occlusal Reconstruction, Tokushima University Graduate School of Biomedical Sciences, Tokushima, Japan. *

**Keywords:** Oral cancer, cancer prevention, trend analysis, health, seeking behaviour, Philippines

## Abstract

**Objective:**

This study analyzed the health-seeking behavior of Filipinos through relative search volume in Google Trends using oral cancer, mouth cancer, tongue cancer, gum cancer, and lip cancer as predetermined search terms.

**Methods:**

Comma-separated values files containing relative search volumes of search trends pertaining to oral cancer from 2009 to 2019 were assessed. Brown-Forsythe one-way ANOVA was used to measure differences of oral cancer between years and among months. Repeated measures two-way ANOVA measured differences among the searches for mouth cancer, tongue cancer, gum cancer, and lip cancer through the years. Time series models were fitted and used to forecast search interests.

**Results:**

The results revealed that interests in oral cancer were significantly higher in 2019 (43.75±5.5, p<0.05) compared to 2009 (29.0 ± 6.7). In terms of months, searches were higher in February (45.0 ± 6.6) compared to May (24.8 ± 3.4, p=0.015), June (25.3 ± 4.4, p=0.020), and December (26.5 ± 4.0, p=0.038). Search interests for gum cancer and lip cancer remained significantly lower from 2011 to 2019, and tongue cancer from 2016 to 2018 but approximated mouth cancer in 2019. The forecast shows mouth cancer (31.67%), tongue cancer (23.75%), and lip cancer (3.83%) fluctuating through the year, while gum cancer (8%) will remain steady in 2020.

**Conclusion:**

Health-seeking behavior through search trends show an increased interest in oral cancer in 2019 and during February. Search interests will fluctuate in 2020, but at the end of the year will decrease for mouth cancer and tongue cancer, will increase for lip cancer, and will remain steady for gum cancer.

## Introduction

One of the common malignancies in developing countries is oral cancer, where squamous cell carcinoma caused by tobacco and alcohol use is the most common manifestation (Jemal et al., 2011; Torre et al., 2015; Bray et al., 2018; Mattiuzzi and Lippi, 2019). There are cultural and social practices that may expose people to oral cancer. This cancer is common in developing countries located in Southeast Asia and its prevalence persists even after migration to developed countries (Avon, 2004). With over 200,000 new cases per year, oral cancer has been established to be a severe public health dilemma in the world (Parkin et al., 1999; Global Burden of Disease Cancer et al., 2017). Worldwide, oral cancer mortality rate (50%) continues to be high even with modern medical treatments due to its late diagnosis. It has caused more deaths than cervical cancer, melanoma, and Hodgkin’s disease (Miller et al., 2019). About two-thirds of mortalities caused by oral cancer (Parkin et al., 2005) and 25 per 100,000 cases were detected in developing countries. The survival rate in 60% of people diagnosed with oral cancer is estimated to be up to five years only (Jemal et al., 2011), and it has not improved since 30 years ago (Lane et al., 2006).

Cancer is the third leading cause of morbidity and mortality in the Philippines, just after communicable diseases and cardiovascular diseases. Concurrently with heart disease, cancer had an increasing trend from 1942 to 1996. Roughly, 75% of all cancers occur after the age of 50 as opposed to 3% that occur at age 14 and younger. It was estimated that one in every 1,800 Filipinos will develop cancer per year due to late diagnosis. The health-seeking behavior of Filipinos shows that those who suffer from cancer seek medical assistance only in the advanced stages of the disease. The oral cavity, along with lung, breast, cervix, liver, prostate, stomach, colon and rectum, and ovary are the leading sites of cancer in the Philippines. Data pertaining to cancer incidence rate presented by the Department of Health-Rizal Cancer Registry (DOH-RCR) and the Philippine Cancer Society Inc.-Manila Cancer Registry (PCSI-MCR) are limited to the 26 municipalities of Rizal Province and the four cities of Quezon, Manila, Caloocan, and Pasay. The earliest study showing the link between betel nut chewing and oral cancer can be traced back as early as 1915, where it was found that 70% of cases were caused by this habit (Ngelangel and Wang, 2002).

The Philippines has a comprehensive cancer prevention plan introduced by the Department of Health – Philippine Cancer Control Program (DOH-PCCP). For instance, community-level primary prevention and hospital-level tertiary prevention are being used as preventive measures by the Lung Cancer Control Program. For breast cancer, a nationwide stratagem against breast cancer is presented by the Breast Cancer Control Program through community health case finding and treatment, public information, and health education. For liver cancer, vaccination of newborns, health checkups among commercial sex workers, prevention of multiple medical syringe use, and public education were implemented against hepatitis B and hepatitis C virus. These viruses are implicated in liver cancer. To prevent oral cancer, the DOH-PCCP encourages dentists and primary care physicians to do regular examinations for cancerous lesions of the mouth and provide counseling to people who smoke cigarettes, chew betel-quid or tobacco, and those who have alcohol abuse (Ngelangel and Wang, 2002).

Prevention is a key factor in the survivability of cancer patients. In the digital age, most of the people, including Filipinos, surf on the Internet to gain information regarding prevention and treatment of diseases. Facebook (Abreo et al., 2019), Twitter (Bautista and Lin, 2015), and Google (Ho et al., 2018) are usually mined for data as an inexpensive tool in analyzing trends in the health-seeking behavior of Internet users. Considering Philippines, the data from the DOH-RCR and PCSI-MCR are limited to the main island of Luzon and not representative of the whole Philippine population. This is where Google Trends becomes an invaluable adjunct in determining the epidemiology of disease and consequentially creating programs for early detection or prevention. Google Trends towards observation of incidence and epidemic (Nuti et al., 2014) have been used exhaustively, especially for diseases such as Ebola (Alicino et al., 2015), Zika (Morsy et al., 2018), Malaria (Ocampo et al., 2013), Influenza (Xu et al., 2017), and Oral problems (Patthi et al., 2017). The relative search volumes were analyzed for trends to determine significant differences regarding the interests of Internet users by using a specified search parameter over a period of time and database .

Using Google Trends, this study’s primary aim was to evaluate the health-seeking behavior of Filipino Internet users in terms of oral cancer. Generally, the objectives of this research were to (1) evaluate the differences in peak popularity rate through studying relative search volume for oral cancer from 2009-2019, using 2009 as the point of comparison; (2) gauge the peak popularity rate of oral cancer per month from 2009-2019; (3) determine the differences in peak popularity rate of the different oral cancer sites from 2009-2019; (4) create a year-long forecasting for the search trends for different cancer sites in 2020; and (5) compare the 2020 projected relative search volume to the 2009 data.

## Materials and Methods

*Search Parameters and Protocol*

The first search query was done using the search topic of “oral cancer”, the Philippines as location, January 2009 to December 2019 as timeline, health as the category, and web search as the database. This was done to gather information regarding the health-seeking behavior or overall interest of Filipinos regarding search on line for oral cancer.

The second search query was done using “mouth cancer”, “tongue cancer”, “gum cancer”, and “lip cancer” as search terms, Philippines as location, January 2009 to December 2019 as timeline, health as the category, and web search as the database. This search strategy was used to ascertain the interests of Internet users on finding data about different cancer sites. The higher the peak popularity rate, the greater the presumed interest of the Filipinos towards the prevention or treatment of specific cancer sites.

Interests over time and compared breakdown by sub-region graphs can be readily observed in the graphic-user interface panels of the Google Trends website. Comma-separated values files relating to this information were downloaded. Data were re-arranged in an array suitable for analyses, and graphs were reconstructed.

*Data Analyses*

Brown-Forsythe one-way ANOVA with post hoc Dunnett’s test was done to determine the difference between years across from 2009 to 2010 in terms of oral cancer search trends. Brown-Forsythe one-way ANOVA with Tukey’s post hoc test was also done to reveal the differences regarding oral cancer searches per month from 2009 to 2019. Repeated-measures two-way ANOVA with Geisser-Greenhouse correction and Dunnett’s multiple comparison test were used to examine the effect of year on the rate of search interests for mouth cancer, tongue cancer, gum cancer, and lip cancer. Trend series modeler was used to determine models and fit them for year-long forecasting of interest from January to December 2020. The projected data were examined and compared with those found for 2009 data using repeated-measures two-way ANOVA with post hoc Dunnett’s test. GraphPad Prism version 8 was used for inferential statistics and SPSS (version 26) was used for model-fitting and forecasting.

## Results

*Search Trends for Oral Cancer*

The top regions of the Philippines that were most interested in searching for oral cancer using Internet, in descending order, were Cordillera Administrative Region (100%), Bicol (97%), Ilocos Region (94%), Calabarzon (87%), Western Visayas (86%), Central Visayas (84%), Metro Manila (84%), Central Luzon (77%), Davao Region (72%), and Northern Mindanao (68%).

**Figure 1:**
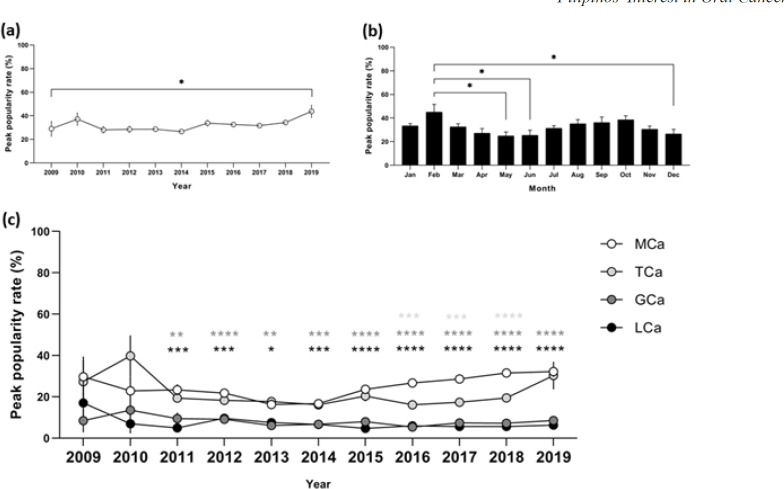
Peak Popularity Rate Percentage of (a) OCa from 2009 to 2019 analyzed yearly showing a significant increase in 2019; (b) OCa from 2009 to 2019 analyzed monthly showing a significant increase in February compared to May, June, and December; (c) MCa, TCa, GCa, and LCa, where GCa and LCa were substantially lower compared to MCa from 2011 to 2019. OCa, oral cancer; MCa, mouth cancer; TCa, tongue cancer; GCa, gum cancer; LCa, lip cancer

Considering the relative search volume for oral cancer across different years, a statistically significant difference between year and the relative search volume was found based on Brown-Forsythe one-way ANOVA test (F (10,121) = 3.793, p<0.001). A Dunnett’s post hoc test revealed that the peak popularity rate percentage in 2019 (43.75 ± 5.5, p<0.05) was significantly higher compared to 2009 (29.0 ± 6.7). In contrast, there was no statistical difference between the peak popularity rate percentage in 2009 and 2010 (p=0.578), 2011 (p=0.999), 2012 (p=0.999), 2013 (p>0.999), 2014 (p=0.999), 2015 (p=0.961), 2016 (p=0.992), 2017 (p=0.999), or 2018 (p=0.931) (Figure 1a).

From 2009-2019, there was a statistically significant difference between relative search volume for oral cancer as determined by Brown-Forsythe one-way ANOVA ) F (11,120) = 2.449, p<0.01). Tukey post hoc test revealed that the peak popularity rate percentages in May (24.8 ± 3.4, p=0.015), June (25.3 ± 4.4, p=0.020), and December (26.5 ± 4.0, p=0.038) were significantly lower compared to that in February May (45.0 ± 6.6). In contrast, there was no statistical difference between February and January (p=0.577), March (p=0.482), April (p=0.054), July (p=0.329), August (p=0.798), September (p=0.895), October (p=0.989), or November (p=0.254) in terms of the peak popularity rate percentage (Figure 1b)

*Specific Cancer Sites Search Trends*

A repeated-measures two-way ANOVA with Geisser-Greenhouse correction was conducted to examine the effect of year on the relative search volume for mouth cancer, tongue cancer, gum cancer, and lip cancer. A statistically significant interaction between the effect of year and the number of searches for mouth cancer, tongue cancer, gum cancer, and lip cancer was detected (F (3, 44) = 68.97, p<0.0001). Dunnett’s multiple comparisons test showed that search rates for gum cancer (p=0.006) and lip cancer (p<0.0001) were significantly lower than that for mouth cancer in 2011; gum cancer (p<0.0001) and lip caner (p=0.001) were significantly lower than mouth cancer in 2012; search rates for gum cancer (p=0.004) and lip caner (p=0.012) were significantly lower than that for mouth cancer in 2013; search rates for gum cancer (p<0.001) and lip cancer (p<0.001) were significantly lower than that for mouth cancer in 2014; search rates for gum cancer (p<0.0001) and lip cancer (p<0.0001) were significantly lower than that for mouth cancer in 2015; search rates for tongue cancer (p<0.001), gum cancer (p<0.0001), and lip cancer (p<0.0001) were significantly lower than that for mouth cancer in 2016; search rates for tongue cancer (p<0.001), gum cancer (p<0.0001), and lip cancer (p<0.0001) were significantly lower than that for mouth cancer in 2017; search rates for tongue cancer (p<0.0001), gum cancer (p<0.0001), and lip cancer (p<0.0001) were significantly lower than that for mouth cancer in 2018; search rates for gum cancer (p<0.0001) and lip cancer (p<0.0001) were significantly lower than that for mouth cancer in 2019 (Figure 1c).

*Cancer Sites Search Trends Forecast*

**Figure 2:**
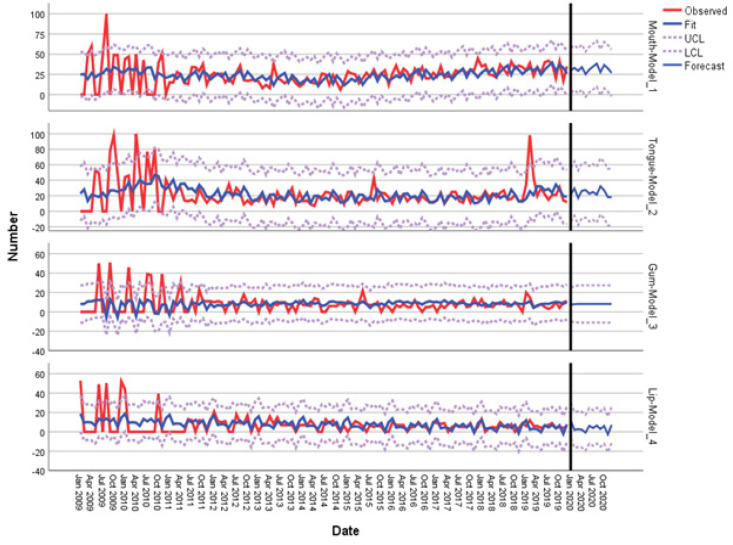
Year-Long Forecasting of MCa, TCa, GCa, and LCa through 2020. MCa, mouth cancer; TCa, tongue cancer; GCa, gum cancer; LCa, lip cancer

Simple seasonal model for mouth cancer and tongue cancer, ARIMA (0,0,2) (0,0,0) for gum cancer, and Winter’s Additive model for lip cancer were fitted to forecast search trends for cancer sites from January to December 2020. The forecast showed that search for mouth cancer (31.67%), tongue cancer (23.75%), and lip cancer (3.83%) would be fluctuated through the year, while that for gum cancer (8%) would remain steady. By the end of 2020, it was estimated that search rate mouth cancer and tongue cancer would decrease, while for lip cancer would increase, and for gum cancer would remain unremitting (Figure 2).

**Figure 3:**
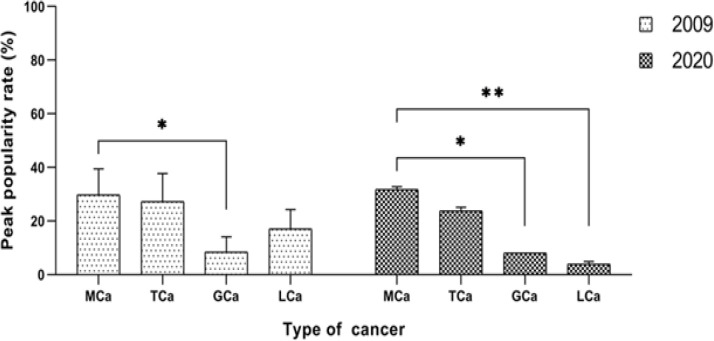
Comparison of Peak Popularity Rates of Each Cancer Site in 2009 and 2020. MCa, mouth cancer; TCa, tongue cancer; GCa, gum cancer; LCa, lip cancer

The difference between 2009 and 2020 concerning the number of searches for mouth cancer, tongue cancer, gum cancer, and lip cancer was investigated using repeated-measures two-way ANOVA. It was found that there was a statistically significant interaction between the effect of year and the number of searches for mouth cancer, tongue cancer, gum cancer, and lip cancer (F (3, 44) = 5.862, p=0.002). Dunnett’s multiple comparisons test showed that search rate for gum cancer (p=0.040) was significantly lower in 2009 and would continue to be lower in 2020 (p=0.019) compared to that for mouth cancer. In contrast, there was no difference in 2009 between lip cancer and mouth cancer, but search rate for lip cancer (p=0.005) was projected to be significantly lower in 2020 (Figure 3).

## Discussion

Cordillera Administrative Region, Ilocos Region, Western Visayas, Central Visayas (84%), Central Luzon, Davao Region, and Northern Mindanao are all Tobacco-producing regions in Philippines, in which there are a high prevalence of cancer (Appau et al., 2019). About 64% of oral cancer in the United States are caused by human papillomavirus contracted through oral sex. Rises in interest in regions like Metro Manila and Central Visayas, that are urban areas with documented sex workers, may be indicative of oral cancer caused by the human papillomavirus or HIV (Urada et al., 2012).

The results of this study revealed that the health-seeking behavior, shown through the search trend for oral cancer, was steady from 2010 to 2018 but increased in 2019. This could be interpreted as an increase in the incidence of oral cancer. However, this finding should be corroborated by comparing it with actual numbers from Philippine Cancer Registries. Unfortunately, the last data in this regard was published in 2015 and therefore can’t be cross-examined in the context of these findings. This database contains data gathered only from 26 municipalities of Rizal Province and the cities of Quezon, Manila, Caloocan, and Pasay. While these areas are densely populated, they are barely representative of the whole population and the other major islands of Visayas and Mindanao have not been considered. According to the 2015 report, the incidence of oral cancer rose from the 15th leading new cases in 2010 to the 11th in 2015 , which is in line with the findings of this study (Figure 1a).

Searches peaked in February. This could be attributed to Purple Feb and the celebration of the National Oral Health Month (Villas, 2016). A nationwide celebration of oral health advocacy and the emphasis of oral health importance is observed every February in the Philippines. By virtue of Republic Act 9484 and a resolution submitted by the Philippine Dental Association Cebu Chapter, Inc., February is aptly named Purple Feb or the equivalent of Pink October. The search rate for oral cancer was then higher during the month of October although it was insignificant compared to other months (Figure 1b). This outcome could be interpreted in the same light as the results found in a recent study done in Malaysia on breast cancer awareness (Mohamad and Kok, 2019). Currently, the dental association does not provide a comprehensive oral cancer prevention program and is not one of the several professional organizations collaborating with the Philippine Cancer Society. The rise in searches could be due to oral health general public education programs provided by the dental association and individual dental care personnel .

As it is clear from Figure 1c, this study uncovered that searching for gum cancer and lip cancer was considerably lower compared to searching for mouth cancer from 2011 to 2019 In addition, it was revealed that searching for tongue cancer was low from 2016 to 2018, but rose in 2019. The comparability of tongue cancer to mouth cancer could be alarming if it will be interpreted as health-seeking behavior due to new cases. This is comparable to the global increase in tongue cancer in developing (Cohen Goldemberg et al., 2018) and developed nations (Haeggblom et al., 2019). This cannot be verified by the data provided by the Philippines cancer registries as all types of oral cancer are grouped into one.

It is estimated that the search trends for mouth cancer, tongue cancer, and lip cancer will continue to fluctuate in 2020, while it will be steady for gum cancer according to the simulated forecast based on the fitted forecast models. Search rate for mouth cancer peaks in July and October, while it peaks in October for tongue cancer. Both search trends eventually decline at the end of the year. The same oscillations with decreased amplitudes and frequencies can be predicted for lip cancer. However contrary to the two aforementioned search trends, search trend for lip cancer rise at the end of the year. The steady and short amplitude search trend for gum cancer can be interpreted as existing gum cancer cases that might eventually treated following the proper treatment (Figures 2 and 3). 

In the Philippines, the most crucial risk factor for oral cancer is aging. Although from 1980 to 2007 its incidence rates declined 2.9% in males and 4.3% in females, oral cancer five-year survival rate was 27% and ten-year survival rate was 17% in 2012. The Philippine Cancer Society and the Department of Health has determined betel nut chewing, inverted cigarette smoking, excessive alcohol consumption, and unbalanced diets to be the other risk factors that increase the likelihood of oral cancer . Prevention of oral cancer is possible by increasing public health education on the causes and preventive measures for oral cancer (Laudico et al., 2015). 

Solving the problem of oral cancer in the Philippines might be possible but it will be challenging. On the other hand, addressing the problem of alcoholism would be difficult as it has been a pervasive, complex, and lasting problem (O’Connor and Ruiz, 2014; Tuliao et al., 2016). There are anti-smoking policies and initiatives that have been done but yet the Philippines remain to be hooked on smoking tobacco. The government should increase its crusade in preventing oral cancer by imposing stricter guidelines and penalties to counter alcoholism (Page and West, 2012; Bilano et al., 2015). Unbalanced diets are also common in the Philippines, which is evidenced by the high onset of diabetes and cardiovascular diseases. Filipino cuisine is high in carbohydrates and healthier alternatives need to be chosen (Fernando, 1989; Tiong et al., 2018). Regular dental visits should be practiced and inculcated in the people. However, this would be difficult as dental services have been currently devalued in the Philippines. In a recent study it was shown that despite the low willingness to pay for dental services by Filipinos, seeking treatment for oral cancer scored the highest by them (Dalanon et al., 2018). The Philippine Dental Association together with the Department of Health, the Philippine Cancer Society, and the local government units should collaborate to stress the importance of oral cancer prevention. Basic educational institutions should fortify public education in cancer prevention and higher educational institutions should improve education pertaining to cancer prevention in the health professions. Cancer prevention should not be only limited to the month-long celebration of Purple February or Pink October but its importance should be instilled and the consequence of its non-compliance be clarified to Filipinos.

*Study Limitations*

Although 9 out of the 10 regions in the Philippines that scored highest in the interest rate were not included in the cancer registries of the Philippines, this study was not without limitations. Actual data from oral cancer cases from these regions need to be taken into account and correlated with these findings. In this study, the relative search volume downloaded from Google Trends was limited to Internet users using the Google search engine. As previously mentioned, Facebook and Twitter can also be data mined for information. Despite this, Google currently comprises around 70% of the web search industry. In the future, this data should be linked to findings presented by the Association of Southeast Nations region and the world.

In conclusion, this study showed that more people are seeking information about oral cancer as shown in the substantial rise of peak popularity rate percentage in oral cancer searches from 2009 to 2019. The data also revealed that these searches are significantly higher in February than in May, June, and December. Web searches for gum cancer and lip cancer remain to be lower in comparison to that for mouth cancer, yet lip cancer approximated mouth cancer in 2019. Curiosity regarding these cancer sites is estimated to fluctuate all through 2020 according to the forecasted trend lines, but search rate for lip cancer has a tendency to rise up at the end of the year. Despite this, searches for both gum cancer and lip cancer are projected to make a sizeable decrease in relation to search for mouth cancer.
